# Host genetic diversity drives variable central nervous system lesion distribution in chronic phase of Theiler’s Murine Encephalomyelitis Virus (TMEV) infection

**DOI:** 10.1371/journal.pone.0256370

**Published:** 2021-08-20

**Authors:** Koedi S. Lawley, Raquel R. Rech, Faith Elenwa, Gang Han, Aracely A. Perez Gomez, Katia Amstalden, C. Jane Welsh, Colin R. Young, David W. Threadgill, Candice L. Brinkmeyer-Langford

**Affiliations:** 1 Department of Veterinary Integrative Biosciences, College Station, TX, United States of America; 2 College of Veterinary Medicine and Biomedical Sciences, College Station, TX, United States of America; 3 Texas A&M University, College Station, TX, United States of America; 4 Department of Veterinary Pathobiology, College Station, TX, United States of America; 5 Department of Epidemiology and Biostatistics, College Station, TX, United States of America; 6 School of Public Health, College Station, TX, United States of America; 7 Texas A&M Institute for Neuroscience, College Station, TX, United States of America; 8 Department of Molecular and Cellular Medicine, College Station, TX, United States of America; Medizinische Universitat Innsbruck, AUSTRIA

## Abstract

Host genetic background is a significant driver of the variability in neurological responses to viral infection. Here, we leverage the genetically diverse Collaborative Cross (CC) mouse resource to better understand how chronic infection by Theiler’s Murine Encephalomyelitis Virus (TMEV) elicits diverse clinical and morphologic changes in the central nervous system (CNS). We characterized the TMEV-induced clinical phenotype responses, and associated lesion distributions in the CNS, in six CC mouse strains over a 90 day infection period. We observed varying degrees of motor impairment in these strains, as measured by delayed righting reflex, paresis, paralysis, seizures, limb clasping, ruffling, and encephalitis phenotypes. All strains developed neuroparenchymal necrosis and mineralization in the brain, primarily localized to the hippocampal regions. Two of the six strains presented with axonal degeneration with myelin loss of the nerve roots in the lumbar spinal cord. Moreover, we statistically correlated lesion distribution with overall frequencies of clinical phenotypes and phenotype progression to better understand how and where TMEV targets the CNS, based on genetic background. Specifically, we assessed lesion distribution in relation to the clinical progression of these phenotypes from early to late TMEV disease, finding significant relationships between progression and lesion distribution. Finally, we identified quantitative trait loci associated with frequency of lesions in a particular brain region, revealing several loci of interest for future study: lysosomal trafficking regulator (*Lyst*) and nidogen 1 (*Nid1*). Together, these results indicate that the genetic background influences the type and severity of clinical phenotypes, phenotypic resilience to TMEV, and the lesion distribution across strains.

## Introduction

Antecedent infections have been suggested as contributing factors to chronic neurological conditions such as multiple sclerosis, epilepsy, Parkinson’s Disease, and amyotrophic lateral sclerosis [1]. It is thought that a series of immunological or inflammatory events catalyzed by infections in children or young adults can result in increased susceptibility to these types of diseases later in life. Moreover, disease phenotypes can vary greatly between individuals in response to the same infectious agent. The severity, prognosis, and clinical presentation of these diseases are influenced in part by a milieu of factors such as age, sex, and other comorbidities. Along with these factors, genetic background is likely a critical driver of infection responses and subsequent neurological disease phenotypes. These disease phenotypes may share some common characteristics which, in turn, contribute to the degree of severity and long-term prognosis of the neuropathology. Furthermore, genetic differences among individuals can influence not only disease onsets and outcomes, but also the efficacy of medical interventions.

Theiler’s Murine Encephalomyelitis Virus (TMEV) provides a classical model for how a single infectious agent can elicit highly diverse neurological phenotypes in mouse strains with different genetic backgrounds. In so-called “susceptible” mouse strains such as SJL/J mice, TMEV causes demyelinating disease [2], while “TMEV-resistant” C57BL/6J mice develop acute seizures following infection that eventually progress to epilepsy [3,4]. Histologically, responses to TMEV have been well characterized in C57BL/6J, which exhibit hippocampal degeneration and seizures, and SJL/J mice, which present with inflammation and demyelination [5,6]. Subsequently, these inbred mouse strains have been widely used as models for epilepsy and multiple sclerosis, respectively.

The strain-specific outcomes to TMEV infection, observed with a small number of inbred mouse strains, highlight the need for animal models with genetic backgrounds that reflect the level of diversity found in humans. Such models should greatly resemble the variability in disease outcomes observed in humans, thus enabling a better understanding of these outcomes and facilitating the discovery of targets for therapeutics. The genetically heterogeneous Collaborative Cross (CC) mouse resource is advantageous in this regard [7,8]. The CC mouse resource was developed by cross-breeding a panel of eight founder strains, including five classical (A/J, C57BL/6J, 129S1/SvImJ, NOD/ShiLtJ, NZO/HlLtJ) and three wild-derived mouse strains (WSB/EiJ, CAST/EiJ, PWK/PhJ). The resulting recombinant inbred CC strains capture 90% of the genetic diversity, and represent novel combinations of alleles, in domesticated mouse species [9]. The CC mouse resource has been used to model variation in pathogenic phenotypes after fungal [10], bacterial [11,12], and viral infections [11,13,14], and is well suited to elucidate the role of genetic background on infectious disease pathophysiology. We have previously used the CC to model the myriad of subtle differences in neurological outcomes to TMEV infection in addition to demyelination [15,16].

For the current work, we have hypothesized that clinical outcomes to TMEV infection–which often resemble human neurological responses to viral infections [15]–are associated with the relative locations in the central nervous system. To test our hypothesis, we have precisely localized lesions in six CC mouse strains selected to represent phenotypic extremes of TMEV outcomes, including three previously described [15] and three novel CC strains. We also evaluated TMEV-induced phenotypes and lesions in two inbred strains commonly used to study TMEV infection (SJL/J and C57BL/6J), to place our findings in context. Rather than simply demonstrating that different genetic backgrounds experience pathological changes in the central nervous system (CNS), as before [16], for the current study we have measured the strain-specific prevalence of various CNS lesions, along with their precise location. Next, we have identified statistical correlations between clinical symptoms (phenotypes) and lesion frequency and location. For this, we evaluated relationships between lesion location and the observational frequencies of neurological disease phenotypes including righting reflex, paresis, paralysis, seizures, limb clasping, ruffling, and encephalitis [15], all defined at the same chronic phase time point. We identified statistical correlations, overall and on a strain-by-strain basis, between these phenotypes and lesion burden at specific locations.

Our findings provide insight into how “susceptibility” to a viral infection ultimately varies in terms of subsequent neuropathology. This knowledge is critical to increasing our understanding about how seemingly similar outcomes can result from different lesion profiles and how the same lesions can be associated with different clinical signs. Our findings also provide a foundation for future studies to reveal the diverse molecular mechanisms contributing to variable neuropathologies. Finally, this work enables us to identify and prioritize CC strains for further characterization and assessment of TMEV pathology, and as potential models for human neurological conditions caused or affected by viral infection.

## Methods

### Mouse care and infection procedure

All animal care protocols were in accordance with NIH Guidelines for Care and Use of Laboratory Animals and were approved by the Texas A&M University Laboratory Animal Care and Use Committee (AUP 2017–0082). Breeding of all CC mice was performed in-house at Texas A&M University.

The mice were maintained in an AAALAC approved facility under 14-h light and 10-h dark cycle with *ad libitum* food and water. Sixty-three mice between 3 and 4 weeks of age, including females and males from the strains SJL/J and C57BL/6J, and six CC lines including one CC-RIX (recombinant inbred intercross), were anesthetized by isoflurane inhalation (MWU, Meridian, ID) and intracerebrally injected into the right mid-parietal cortex (approximately 1.5 mm ventral) with 5.0x10^4 plaque forming units (PFU) of the BeAn strain of TMEV (American Type Culture Collection [ATCC] VR 995, Manassas, VA) in 20 μl of phosphate buffered saline (PBS). Sham infected mice (n = 46) were anesthetized and intracerebrally injected with PBS only. Mice were randomized to treatment. Mice were housed 4–5 to a cage and observed and weighed daily to monitor general health. Measures were taken to minimize mouse pain and stress including the provision of softened food pellets. Mice which lost more than 20% of their pre-infection body weight prior to the 90 dpi endpoint were euthanized and excluded from the study.

### Clinical progression phenotyping and scoring

All mice were assessed using established methods for evaluating TMEV-induced neurological disease phenotypes and sickness [15]. Mice were assessed twice daily for clinical signs from 0-14dpi (acute phase). Following the acute phase all mice were given weekly scores. Phenotypes scored included delayed righting reflex, paresis, paralysis, seizures, limb clasping, encephalitis, and ruffling. The *90 dpi clinical phenotype frequency* of each measure (reflex, paralysis, paresis, seizures, limb clasping, encephalitis, ruffling) was calculated as the number of observations of that particular phenotype for each strain, divided by the total number of observations by that time point. The overall *progression scores* were calculated as described previously [15], using the difference between cumulative phenotypic frequencies from 14 dpi to 90 dpi, to better define “resilience” to TMEV and to understand the relative trajectory of each phenotype (e.g., symptom improvement or worsening) over the course of infection.

#### Delayed righting reflex

We assessed delayed righting reflex by turning each mouse on its back on a flat surface and recording the time to right itself to a prone position, with all four paws underneath it, for at least two trials. A delay in righting reflex is suggestive of dysfunction in vestibular pathways, spinal interneurons, proprioceptive afferents, and motor neurons [17,18].

#### Paresis and paralysis

Mice were observed for signs of paresis or paralysis when walking on a flat surface, and when placed on a metal grate and inverted [19]. Mice were allowed to navigate the grate, and each of the limbs were scored on the following scale: 0 –mouse was active and able to walk with no signs of weakness; 1 –limb dangled from the grate for several seconds before recovery; 2 –limb dangled off of grate more than 50% of the time; 3 –limb continuously dangled from grate (due to paralysis or paresis); 4 –mouse was unable to stay on grate due to whole body weakness (in this case, every limb would receive a score of 4). A limb retaining limited movement was scored as having paresis. If a mouse was completely unable to grip the grate and/or was not moving when walking on a surface, the limb was scored for paralysis.

#### Seizures and limb clasping

Seizures and limb clasping phenotypes were classified by the presence of limb clasping and/or seizures as we have described previously (note that limb clasping was previously referred to as “clonus;”[15]). Limb clasping was scored as follows: 0 –no limb clasping; 1 –limb clasping [20]. Additional details regarding strain-specific seizures and limb clasping phenotypes observed in CC mice, including time points and severity, have been described previously [15]. Therefore, in the present study we only denoted the presence or absence of seizures and limb clasping.

#### Encephalitis and ruffling

Sickness phenotypes recorded included piloerection (ruffled fur) and encephalitis [21–24]. For piloerection, a score of 1 was assigned if the mouse appeared ruffled and if sham-infected mice of the same sex and strain were not similarly ruffled. For encephalitis, a score of 1 was assigned when ptosis was present, again in relation to the appearance of sham-infected mice of the same sex and strain. Conversely, a score of 0 was assigned for a normal appearance for both piloerection and encephalitis.

### Euthanasia and tissue collection

Mice were euthanized between 87–94 dpi via intraperitoneal (i.p.) injection of a lethal dose of Beuthanasia -D Special 150 mg/kg (Schering-Plough Animal Health) as previously described [25]. Mice were transcardially perfused through the left ventricle with 10 mL of ice-cold phosphate buffered saline. Following perfusion, necropsy was performed on each mouse, and one cerebral hemisphere and the spinal cord was collected and fixed for at least 48 hours in 10% formalin.

### Histological evaluation of central nervous system

Coronal sections of the brains for all mice were collected at 4 different levels (as initially defined by [26], with modifications to level names): level A (frontoparietal cortex, septal nuclei and caudate-putamen, nucleus accumbens), level B (frontoparietal cortex, hippocampus [CA regions—1, 2, 3, dentate gyrus], and thalamus), level C (occipital cortex and rostral colliculi of the midbrain) and level D (cerebellum, cerebellar peduncles and pons). Additionally, transverse sections were collected from cervical, thoracic, and lumbar spinal cord. All sections were processed, embedded in paraffin wax, and stained with hematoxylin and eosin (H&E). Spinal cord sections from one CC002 and one CC023 mouse were stained with Luxol fast blue-PASH. All slides were reviewed by a board-certified veterinary pathologist, blinded to slide identity, using an Olympus BX43-F microscope at 40X magnification with a DP73 camera, ND filters and CellSens Standard Software. All sections of the brain were also scanned at 20X and viewed using ImageScope (Aperio Technologies, Vista, CA) to map, trace, and transpose onto schematics using the Allen Mouse Brain Atlas, and Adobe Illustrator (Adobe Systems) for lesion location comparison within and across mouse strains. All histological evaluations were performed by an experimenter blinded to the treatment of the animals.

### Immunohistochemistry

To evaluate the axonal changes in nerve root lesions in the lumbar spinal cord segment, we selected a CC002 mouse and a CC023 mouse for neurofilament immunohistochemistry. No antigen retrieval was performed. Endogenous peroxidase activity was blocked using 3% hydrogen peroxide (Fisher H324-500) for 5 minutes. Universal Blocking Reagent 10X (Power Block) (BioGenex HK085-5K) was applied for 5 minutes. Sections were then incubated for 60 minutes with the anti-neurofilament, clone NE-14 (BioGenex MU073-UC [1:1000]) followed by a 10 minute incubation with biotinylated anti-mouse (Vector BA-9200, 1:100). Detection was performed with 4+ Streptavidin HRP Label (Bio Care Medical, Ap604H) for 10 minutes and Beazoid DAB Chromagen Kit (Bio Care Medical, BDB2004L) for 12 minutes.

### Genetic association analysis

We calculated the frequency of lesion presence for each CC strain as the number of mice with a lesion observed in a given location, divided by the total number of mice analyzed for that strain. To identify genomic regions associated with the calculated lesion frequency, we used the gQTL online software platform [27], which considered the genomes of CC002, CC023, CC027, CC057 and CC078. Significance thresholds were determined using 1000 permutations and p-value of less than 0.05.

### Statistical analysis

The frequency of clinical signs, including reflex, paralysis, paresis, limb clasping, seizures, encephalitis, and ruffling, were measured across 14 and 90 dpi. Analysis of variance (ANOVA) was used to test the association between the progression score of each clinical phenotype (i.e., difference between clinical phenotype score at 90 and 14 dpi) and strains SJL/J, C57BL/6J, CC002, CC012xCC032, CC023, CC027, CC057, and CC078. Similarly, ANOVA was used to test the association between each clinical phenotype outcome at 90 dpi and strain. For each lesion distribution variable (i.e., brain level A through D, cervical, thoracic, lumbar spinal cord segments), Fisher’s exact test was used to test the association between lesion distribution and strain. Moreover, within each strain, Wilcoxon rank-sum test was used to test if each brain level and spinal cord segment was significantly associated with the clinical phenotypes. Association between sex and each phenotype was tested using Wilcoxon rank-sum test within each strain, and the association between sex and each brain level and spinal cord segment was tested using Fisher’s exact test. The Bonferroni-Holm method was used to address the issue of multiple comparisons. A p-value ≤0.05 was considered statistically significant, while p-values between 0.05 and 0.1 were considered suggestive or marginally significant. All analyses were conducted with Statistical Analysis System (SAS) software, version 9.4 (SAS Institute, Cary, NC).

## Results

### Clinical phenotypes are driven by infection status and genetic background at 90 dpi

To later relate the phenotypes to the lesion distribution and identify similarities between strains, we evaluated cumulative clinical phenotype frequencies over the course of 90 days for both sham and infected mice of each strain. We detected significant differences between the presence of TMEV-induced clinical phenotypes between sham and infected mice (S1 Fig). Additionally, we evaluated sex differences in the infected individuals based on previous findings [15]. There were not enough mice of both sexes in every strain for drawing reliable conclusions about sex differences; however, we did not observe any trends suggesting sex-specific differences in the cumulative clinical phenotype frequency at 90 dpi (S2 Fig).

**Fig 1:**
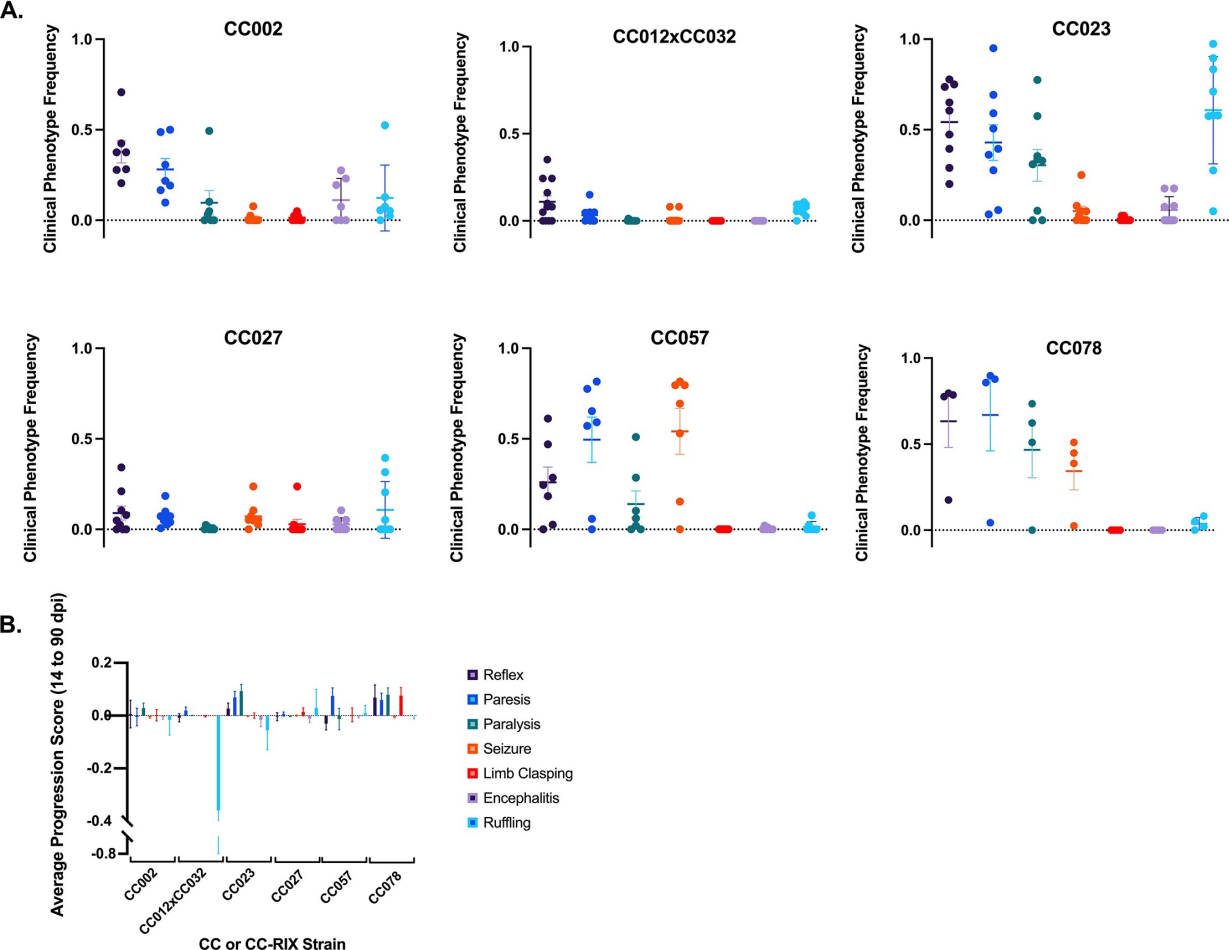
Cumulative frequency and progression scores of clinical phenotypes. a) Cumulative frequency of neurological and sickness phenotypes, based on observations over a 90-day period, varied by strain. Phenotypic observation frequencies for righting reflex, paresis, paralysis, seizures, limb clasping, encephalitis, and ruffling were recorded over the 90 day infection period. Data shown here are mean ± SEM of the average clinical phenotype frequency across 90 dpi for each strain. b) Clinical progression of neurological and sickness phenotypes varied by strain. Phenotypic observation frequencies for righting reflex, paresis, paralysis, seizures, limb clasping, encephalitis, and ruffling were calculated at 14dpi and 90 dpi and the differences from 14 to 90dpi were compared to generate a progression score to understand the effect of the virus during the chronic phase of infection. For each phenotype, positive progression scores indicate that severity increased over time. Data shown here are mean ± SEM of the progression score for each strain.

Overall, the infected strains CC002 and CC023 shared a phenotypic profile with the most prominent phenotypes being increasingly delayed righting reflex, along with paresis and ruffling. CC057 and CC078 mice also often exhibited delayed righting reflex and paresis, along with seizures; paresis was the predominant phenotype for this profile based on overall frequency. The profile shared by CC012xCC032 and CC027 featured low frequencies for all phenotypes (Fig 1A). Cumulative clinical phenotype profiles for SJL/J and C57BL/6J mice are provided in (S1–S3 Figs).

### Genetic background significantly influences multiple aspects of the clinical progression of neurological disease

To better understand the progression clinical phenotypes and the resilience of strains to TMEV between the late acute phase and chronic phase, we calculated observation frequencies of delayed righting reflex, seizures, limb clasping, paresis, paralysis, ruffling, and encephalitis phenotypes between the late acute (14 dpi) and chronic phase of infection (Figs 1B and S4). We detected significant differences in progression score between sham and infected mice (S5 Fig). We detected no significant sex differences in clinical progression score for any phenotype (S6 Fig).

We previously used RNA sequencing to evaluate relationships between TMEV persistence/clearance and disease progression or clinical phenotypes [15]. In that study, we determined that levels of TMEV RNA at 90 dpi do not correlate with disease progression or clinical phenotypes in CC strains, including strains used for the current study. For example, in that study the highest levels of TMEV RNA still present at 90 dpi were measured in mice of the relatively mildly affected strain CC027 [15].

### Genetic background significantly influenced lesion distribution following TMEV infection

We tested for associations between strain and lesion distribution at specific locations within the brain and spinal cord. Histological evaluation identified lesions in infected mice, but not in sham-infected mice, for all strains. We identified lesions at levels A, B and C of the brain, but not level D. We found a statistically significant association between strain and lesion presence at brain level 3 (FET; 0.017), and between strain and lesion presence at the lumbar spinal cord (FET; <0.0001). Associations were not statistically significant between strain and lesion presence at brain levels A (FET; 0.71) or C (FET; 0.29), or cervical (FET; 0.72) and thoracic spinal cord segments (FET; 0.30).

### Distribution of CNS lesions correlate with cumulative frequencies of clinical phenotypes over 90 dpi

**Fig 2:**
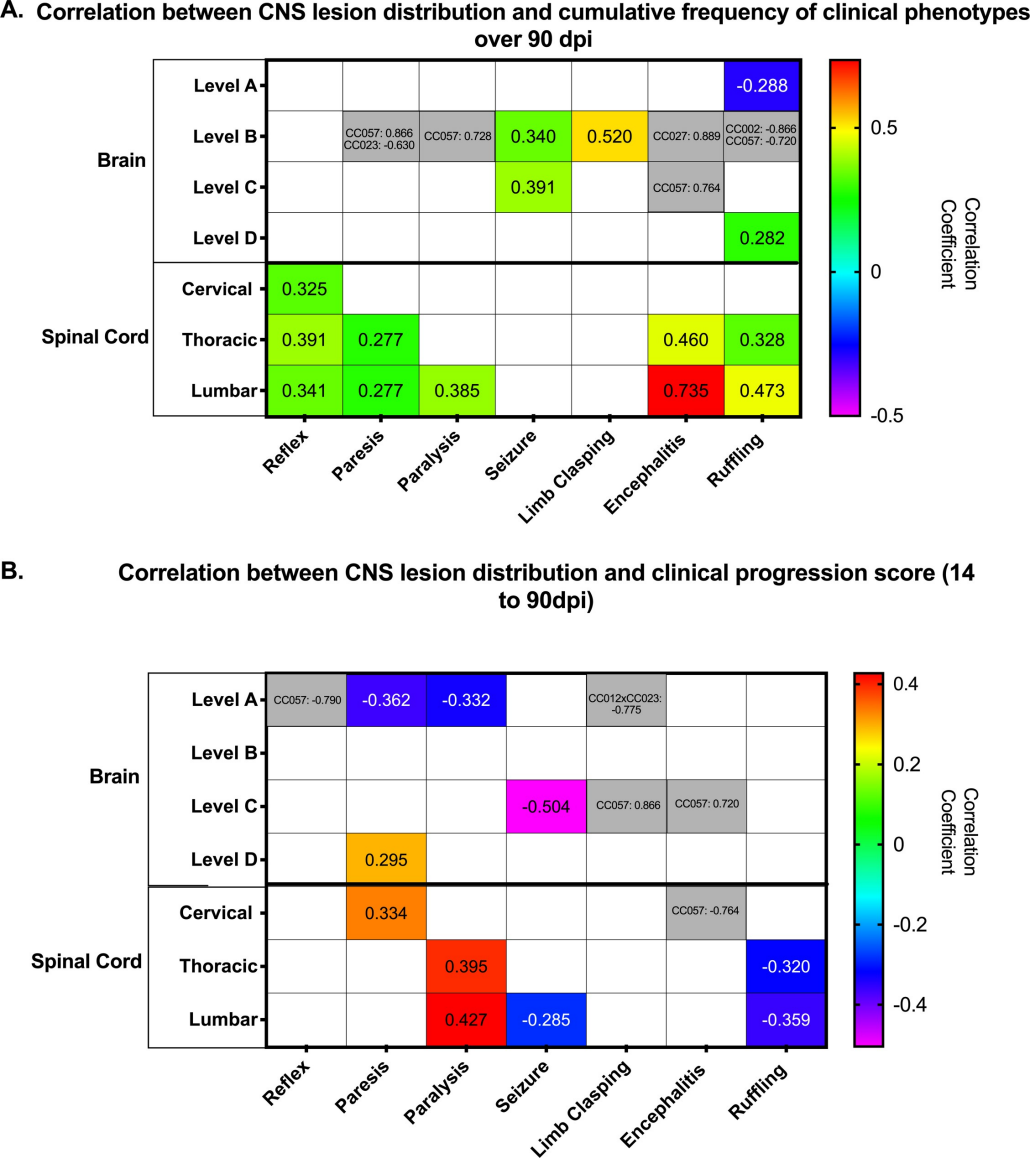
Relationship between 90 dpi cumulative phenotype frequency, progression score and lesion location. a.) Statistically significant associations and suggestive associations between lesion location and 90 dpi phenotypes. b.) Statistically significant associations and suggestive associations between lesion locations and clinical progression scores. The values presented are the Spearman correlation coefficients. The values shown in gray are for strain-specific associations.

We investigated potential relationships between lesion location and cumulative measurement values for phenotypes recorded over 90 dpi (Fig 2A). We identified a marginally significant association between lesions located at brain level A and ruffling observed over 90dpi (ANOVA; p = 0.08 overall; CC027, p = 0.06). We also found significant associations with lesions at brain level B and the cumulative scores for ruffling (CC002, p = 0.01; CC057, p = 0.07), encephalitis (CC027, p = 0.04), seizure (ANOVA; p = 0.04) and limb clasping (p = 0.001), with a trend towards significant for CC057 (ANOVA; paresis, p = 0.052) and CC002 (ANOVA; strain CC002; p = 0.052). For brain level C, we detected a significant association with paresis (ANOVA; p = 0.04) and seizures (p = 0.02; CC002, p = 0.07), and a trend towards significance for CC057 (ANOVA; encephalitis, p = 0.07). Furthermore, spinal cord lesions were significantly correlated with ruffling (ANOVA; thoracic, p = 0.05 overall and CC023 p = 0.09; lumbar, p = 0.003) as well as delayed righting reflex (ANOVA; cervical, p = 0.02; lumbar, p = 0.04) and paralysis phenotypes (ANOVA; thoracic, p = 0.05). Cervical spinal cord lesions were nearly significantly associated with encephalitis in CC057 (p = 0.05).

### Locations of CNS lesions correlate with progression of neurological phenotypes

To identify relationships between lesion distribution and progression of clinical disease phenotypes between the acute and chronic phases of the infection, we statistically evaluated the strength of connections between progression scores and lesion locations (Fig 2B). We found a marginally significant association between lesions at level A of the brain and progression scores for paralysis (ANOVA; p = 0.07), delayed righting reflex for CC057 (ANOVA; p = 0.08), and limb clasping for CC012xCC032 (p = 0.07). There was not a significant relationship between phenotypic progression scores and lesions at level B of the brain. However, for lesions at brain level C, we observed a significant relationship with seizures (ANOVA; p = 0.002; strain CC002, p = 0.07) and limb clasping in CC057 mice (p = 0.01), plus near-significant associations with progression scores for paresis (ANOVA; p = 0.10) and encephalitis (strain CC057, p = 0.07). There was also a slight association between lesions at brain level D and paresis (ANOVA; p = 0.08). We found cervical spinal cord lesions to be significantly associated with paresis progression score (p = 0.03) and nearly significantly associated with encephalitis in strain CC057 (p = 0.05). Thoracic spinal cord lesions were significantly correlated with paralysis (ANOVA; p = 0.01). Finally, lumbar spinal cord lesions were very significantly associated with paralysis (ANOVA; p = 0.02) and ruffling progression scores (ANOVA; p = 0.03), with a near-significant in association with seizures (p = 0.09).

### Lesion distribution varied by strain at 90 dpi in TMEV infected mice

#### Brain

**Fig 3:**
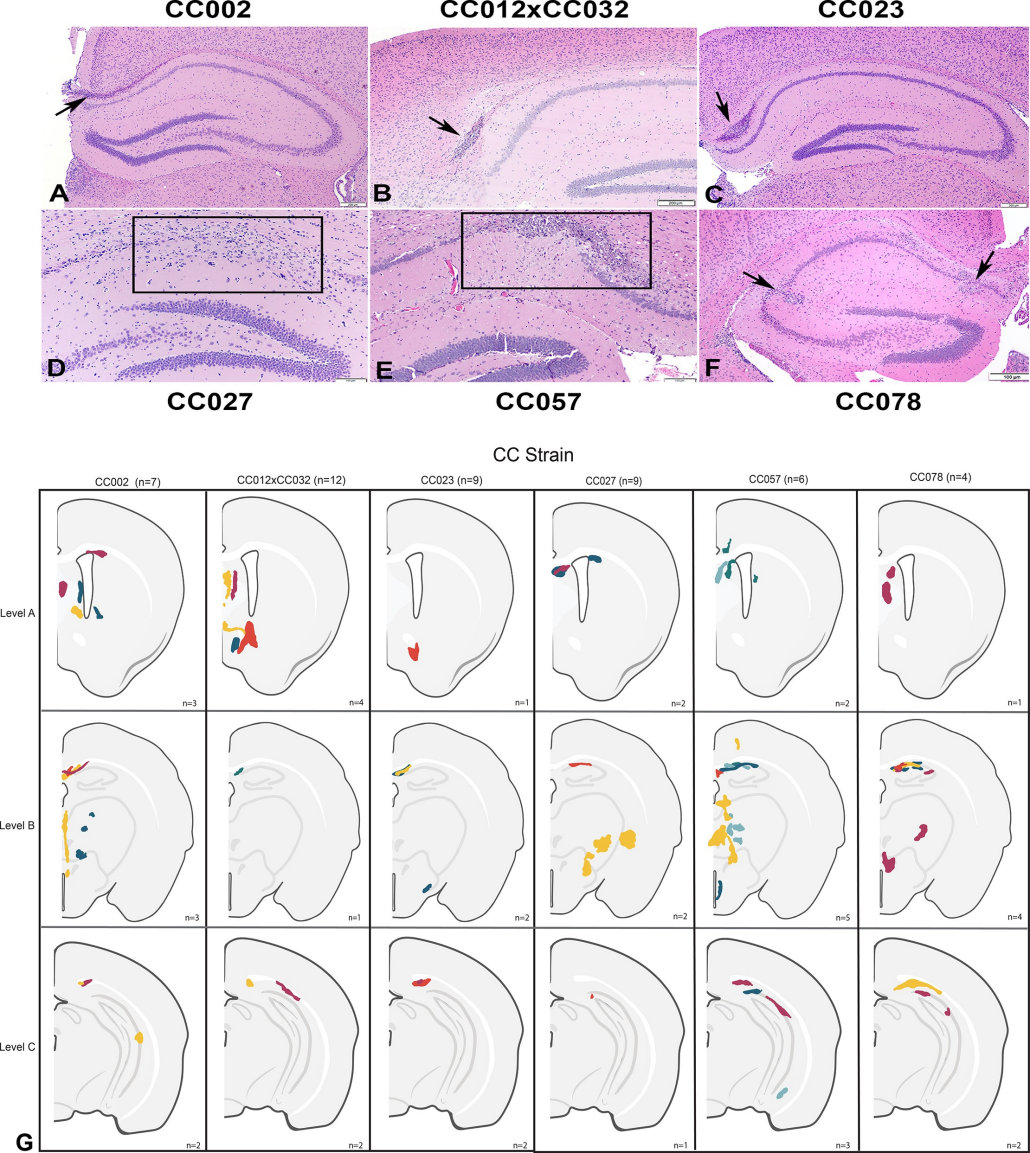
Cross-sections of the cerebrum at level B of CC mice infected with Theiler’s Murine Encephalomyelitis Virus (TMEV) and euthanized ~90 dpi. A. Strain CC002, B. Strain CC012xCC032, and C. Strain CC023: Linear neuroparenchymal necrosis and mineralization of the dorsal hippocampal commissure (black arrows). Hematoxylin and eosin stain; bar = 200 μm. D. Strain CC027: Locally extensive neuroparenchymal necrosis and loss of the hippocampal neurons of the pyramidal layer of CA1 with gliosis of the stratum oriens and stratum radiatum (black rectangle); Hematoxylin and eosin stain; bar = 100 μm. E. CC057 strain: Locally extensive neuroparenchymal necrosis and mineralization of the pyramidal layer of CA1 field with gliosis of the stratum oriens, stratum radiatum and stratum lacunosum-moleculare (black rectangle); Hematoxylin and eosin stain; bar = 100 μm. F. Strain CC078: Focal areas of neuroparenchymal necrosis and mineralization with neuronal loss of the hippocampal CA1 and CA2 pyramidal layer (arrows) and gliosis and mineralization of the superjacent stratum oriens and subjacent stratum radiatum. Hematoxylin and eosin stain; bar = 100 μm. G. Schematic representations of the TMEV-induced lesion distribution in CC strains at brain levels A, B, and C. Each color within the strain column is representative of one animal of the strain.

The main histological findings across all strains examined were multifocal areas of neuroparenchymal necrosis and mineralization with neuronal loss. The lesions were concentrated in the hippocampal formation with adjacent areas, and striatum in all strains (Table 1). The heaviest distribution of these lesions occurred primarily in Field CA1 (including the CA1 pyramidal cell layer and the suprajacent stratum oriens, and subjacent stratum radiatum and stratum lacunosum-moleculare), and suprajacent to field CA1 in the dorsal hippocampal commissure and alveus for CC027, CC057, and CC078. CC002, CC012xCC032 and CC023 also had lesions suprajacent to field CA1, in the dorsal hippocampal commissure and alveus. Fields CA2 and CA3 and the adjacent dorsal hippocampal commissure and alveus were also affected, but to a lesser extent in CC002, CC057, and CC078 (Fig 3 and Table 1).

**Table 1:** Numbers of TMEV-infected CC mice (+) and lesion distributions in the brain.

		Brain									
		Striatum				Hippocampal Formation					
Mouse Strain	Infected	Caudate-Putamen	Septal Nuclei	Nucleus Accumbens	Thalamus	*Field CA1	*Field CA2		*Field CA3		†Adj.
CC002	7	+	+++	-	++	-		+	+		+++
CC012xCC032	12	-	++	+++	-	-		-	-		++
CC023	9	-	-	+	-	-		-	-		++++
CC027	9	-	++	-	+	+		-	-		-
CC057	7	+	++	-	++	+++		+	-		++
CC078	4	-	+	-	+	++++		++	+		-

*Field includes stratum oriens, pyramidal layer, stratum radiatum, and stratum lacunosum-moleculare

**†Adj**; adjacent regions of the hippocampus (dorsal hippocampal commissure and alveus)

**+** indicates the number of infected mice with lesions at given neuroanatomical location,—no lesions present.

The striatum, specifically the septal nuclei, was the second most affected region with neuroparenchymal necrosis and mineralization in all strains except CC023. Strains CC002 and CC057 included one mouse each with lesions in the caudate-putamen region. CC012xCC032 and CC023 had three mice and one mouse, respectively, with lesions in the nucleus accumbens. Six mice across four strains (CC002, CC027, CC057, and CC078) had multifocal to coalescing areas of necrosis and mineralization in the thalamus. In CC023, hydrocephalus was observed in all infected mice and in two out of six sham-infected mice.

### Spinal cord

**Fig 4:**
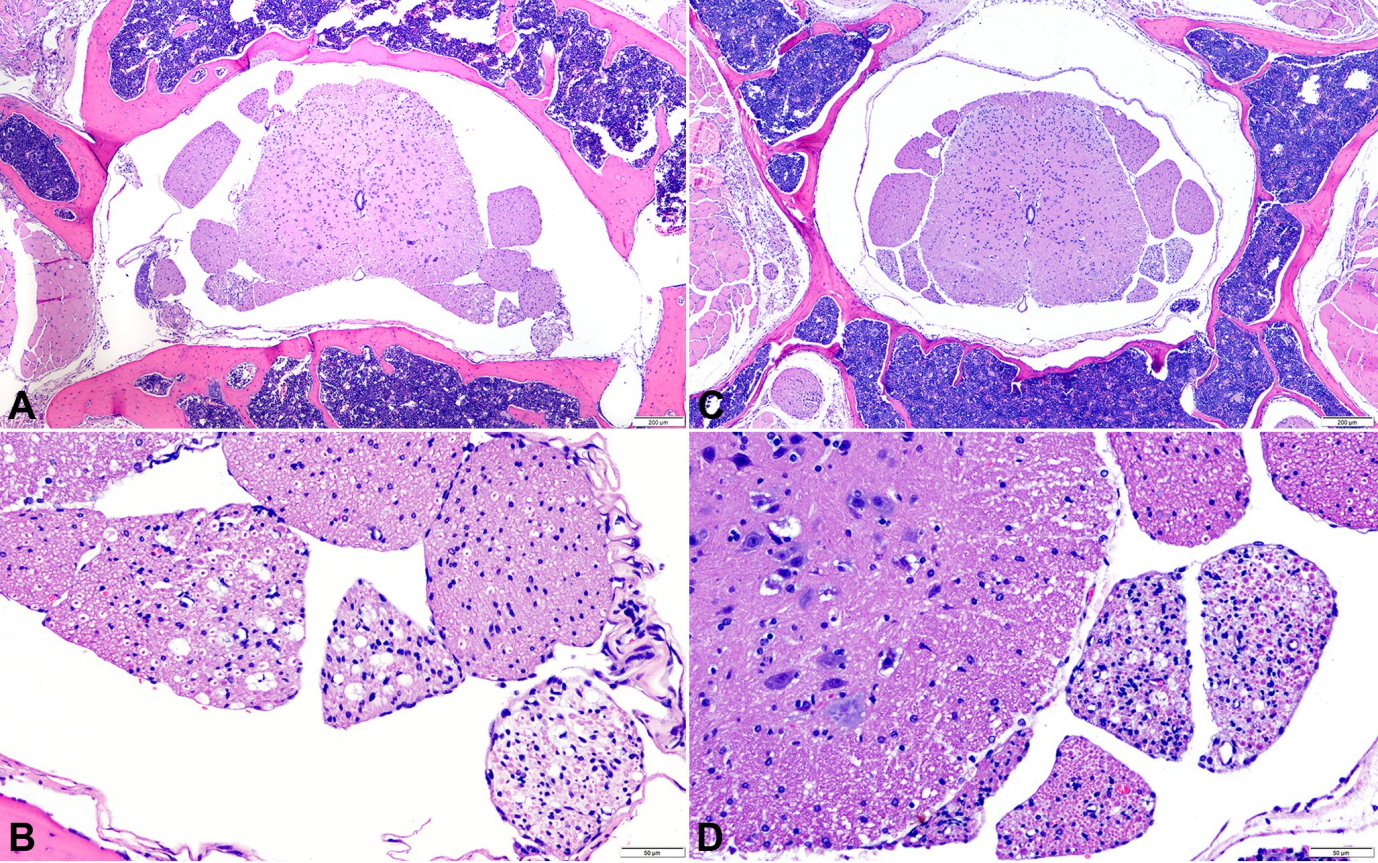
Cross-sections of the lumbosacral spinal cord of CC mice strains infected with Theiler’s Murine Encephalomyelitis Virus (TMEV) and euthanized at ~90 dpi. A. Strain CC002: Bilateral radiculoneuropathy of the ventral nerve roots. Hematoxylin and eosin stain; bar = 200 μm. B. Detail of the lesion shown in A: Chronic axonal degeneration and loss with vacuolation of the myelin sheaths. Hematoxylin and eosin stain; bar = 50 μm. C. Strain CC023: Bilateral radiculoneuropathy of the ventral nerve roots. Hematoxylin and eosin stain; bar = 200 μm. D. Detail of the lesion shown in C: Chronic axonal degeneration loss of myelin sheaths and infiltration of macrophages. Hematoxylin and eosin stain; bar = 50 μm.

As opposed to the histologic findings in the brain, CC012xCC032, CC027, and CC078 had no lesions in the three examined segments of the spinal cord. When present, lesions were mostly located in the lumbar spinal cord which consisted of unilateral to bilateral chronic radiculoneuropathy characterized by axonal degeneration and myelin loss with mild to moderate vacuolation and infiltration of myelinophages (Figs 4 and S10) in all infected CC002 (n = 6/6) and CC023 (n = 5/9). The surrounding skeletal myofibers of these affected mice exhibited marked atrophy. Only one infected mouse, from CC057, had unilateral mild demyelination and gliosis with lymphocytic meningomyelitis in the ventral funiculus of the cervical spinal cord.

### Hippocampal adjacent lesions are associated with several loci of interest

To identify potential quantitative trait loci (QTL) associated with lesion locations, we calculated the relative frequency of lesion presence at the brain and spinal cord regions listed above, as well as specific structures in the brain: from level A, caudate-putamen, septal nuclei, and nucleus accumbens; from level B, thalamus and hippocampal subregions CA1, CA2, and CA3; and hippocampus adjacent regions including dorsal hippocampal commissure and alveus. We used gQTL to identify genomic regions connected to these lesion locations (S7 Fig). Despite our small sample size, we detected a statistically significant association (p = 6.58 x 10^−82^) between relative frequency of lesions in hippocampal adjacent regions, and a locus on chromosome 13 between base positions 13472643–13654592 (mouse genome build 38, mm10). Of the 4 loci located in this region, lysosomal trafficking regulator (*Lyst*) and nidogen 1 (*Nid1*) may be candidates for further study.

## Discussion

In this study, we demonstrated how adolescent viral infections may predispose individuals to develop chronic CNS lesions, which may collectively contribute to the clinical symptoms of neurological diseases based on the genetic background of the host. Our previous work revealed a wide spectrum of neurological disease symptoms in different CC mouse strains following TMEV infection, indicating that the CC population could reveal novel models of diseases in humans by modeling the diverse physiological characteristics of these conditions [15].

Viral infection can often precede by decades and result in highly variable neurological outcomes ranging from no effect to severe clinical signs [28]. Neurological complications, such as encephalitis, multiple sclerosis, and Guillain-Barré syndrome, have been reported following infection with Herpes Virus 6A (HHV-6A) [28], Epstein-Barr virus [28], Zika [29], rabies virus [28], and, more recently, SARS-CoV-2 [30]. Along with the immediate effects of viral infection, viruses have been shown to irreversibly disrupt the structure and function of the CNS via immune-mediated responses. However, responses to the same viral agent can differ drastically depending on the host, and the subsequent clinical signs and prognoses vary.

TMEV infection as a model for neurological diseases has proven valuable for describing severe virally induced neurological sequelae (e.g., demyelination in SJL/J mice, or seizures in C57BL/6J mice), and for better understanding the underlying physiological changes to the nervous system. Previous studies of TMEV-induced neurological dysfunction have typically used inbred mouse strains, and often focused on a narrow range of phenotypes and brain regions. Lesion locations, and their effects, vary somewhat between the commonly studied SJL/J and C57BL/6 strains. During the acute phase of infection (~7 dpi), TMEV infects neurons in the brains of SJL/J mice, and induces polioencephalitis with apoptosis of neurons [31], and axonal damage [32]. The lesion distribution in SJL/J mice extends to the spinal cord as the virus is transported via the axons to the white matter of the spinal cord during the chronic phase [33–36]. Prominent lesions have been observed within the cervical and thoracic spinal cord segments lesions in SJL/J mice [6,36–39]. Meanwhile, TMEV-infected C57BL/6 mice exibit lesions in hippocampal CA1 and CA2 pyramidal cells, periventricular thalamic nuclei, and septal nuclei, which are all areas targeted by TMEV [40].

While discoveries from studies using inbred mice have been critical to our understanding of the roles of viral infection in neurological conditions, the findings are limited in applicability and relevance to humans. By contrast, we used the genetically diverse CC mouse resource and a panel of phenotypes traditionally used to evaluate clinical features of MS, Parkinson’s Disease, epilepsy, ALS, encephalitis, and generalized sickness behaviors associated with infection. In doing so, we have been able to record a much broader spectrum of neurological outcomes following TMEV infection. By evaluating the progression of subtle endophenotypes (e.g., limb clasping) along with more obvious neurological deficits (e.g., paralysis), we developed a complex phenotype profile for different mouse strains [15]. This profile, including the progression score of each of the measured phenotypes, provides context to help explain the complicated relationship between CNS damage resulting after viral infection, and visible signs of neurological disease. Indeed, because of the genetic diversity represented by the CC strains evaluated in this study, we have been able to demonstrate how hosts from diverse backgrounds respond differently to infection by the same virus in terms of neurological disease/clinical symptoms and progression. This information is critical to understanding the wide spectrum of neurological deficits seen in people following viral infections and will form the basis for further studies investigating the genetic contributors to these different phenotypes.

In the present study, all strains of mice infected with TMEV developed neuroparenchymal necrosis and mineralization in the brain, which included areas of the striatum, thalamus, hippocampus and hippocampal adjacent regions. This mineralization is likely associated with inflammation, which may persist months after necrosis subsides [41]. We also observed chronic lesions located at the same neuroanatomic locations as those previously described in acute phase studies in C57BL/6J mice [35,40]. However, we did not observe lesions in piriform, parietal, and entorhinal cortices, as previously described for TMEV in traditional inbred strains.

Three of the six strains analyzed in the current study (CC002, CC023, and CC057) developed lesions in both the brain and spinal cord at similar locations. We observed lesions primarily in the nerve roots of the lumbar segment of the spinal cord in CC002 and CC023 in the chronic phase of the disease, consisting of axonal degeneration with myelin loss. Both the central and peripheral nervous systems were affected in CC002 and CC023 mice, with bilateral radiculoneuropathy of the ventral nerve roots, as well as myofiber atrophy, likely secondary to loss of innervation. Many studies have described the compartmentalization of immune responses between the CNS and peripheral nervous system in infections and inflammatory diseases due to a variety of factors including blood-brain barrier and blood-spinal cord barrier differences and patterns of reactivity in microglia/macrophages [42–46]. However, spontaneously occurring or induced acute inflammatory demyelinating and chronic inflammatory peripheral neuropathies are typically studied using genetic or autoimmune animal models. To the best of our knowledge, only a few studies implement viral infection (e.g., Gallid herpes virus or TMEV) as a driver [47–49]. Viral-induced myositis has been previously observed in different strains of mice infected with TMEV intraperitoneally [50,51]. In the context of TMEV, peripheral nerve lesions develop with intraspinal infection or sciatic nerve injection, rather than the intracerebral infection method used in the present study [47,52]. Interestingly, the symptoms observed for some of these models resemble Guillain-Barre syndrome (GBS) [28,53]. GBS has several subtypes which are characterized by demyelination of the PNS followed by neurological impairment. The observed peripheral nerve root lesions in these two strains of mice appear to share features of GBS such as axonal damage, inflammation, and demyelination, and thus may be a potential model to understand virus mediated PNS lesions. However, further characterization of the clinical symptoms and lesions of these mouse strains is necessary.

Lesion locations were statistically correlated with individual phenotypes based on phenotype scores, but the degree to which any specific lesion location impacted overall phenotypic profile varied. The presence of lumbar lesions had the greatest influence on neurological sequelae. Hippocampal regions were also correlated with clinical phenotype, corroborating with previous TMEV studies [16,54]. However, significant phenotypic correlation to other brain regions indicated a wider range of TMEV-induced damage than previously described.

There were three general profiles identified based on cumulative phenotype frequencies at 90dpi. These profiles can be broadly described by the predominant phenotype for each: “delayed reflex”, “paresis”, and “resilience”. The “delayed reflex” group (CC002 and CC023) featured phenotypes which were statistically correlated with the spinal cord; brain lesions were also significant to phenotypes but somewhat secondary. Conversely, the “paresis” profile (CC057 and CC078) could be considered as being driven primarily by lesions in the brain, followed by spinal cord lesions. Most novel is the “resilience” profile (CC012xCC032 and CC027). These mice developed lesions, but their clinical symptoms were relatively mild. “Resilience” to TMEV has historically not been evaluated; rather, inbred strains have typically been considered “susceptible” or “resistant” in terms of demyelinating disease, and/or viral persistence. One advantage of using CC mice has been the identification of such “resilient” strains, which possess an intriguing ability to sustain neurological damage (lesions) while exhibiting minimal neurological deficits/dysfunction.

Strains CC027, CC057, CC078 all had lesions in the thalamus and CA1 region of the hippocampus, part of brain level B that was significantly associated with seizures and limb clasping at 90dpi. However, CC057 and CC078 had far higher scores for the seizure and limb clasping phenotypes at 90dpi, and more lesions recorded in brain level B, compared to “resilient” strain CC027. The ruffling phenotype score at 90dpi, on the other hand, was nearly significantly associated with brain level A (including septal nuclei, caudate-putamen, and nucleus accumbens). CC002 and CC023, both of the “delayed reflex” profile, and CC012xCC032 of the “resilient” profile all had lesions in these areas. Despite having more lesions at brain level A, CC012xCC032 ruffling and encephalitis scores were much lower. It is possible that lesions found in adjacent regions of the hippocampus (dorsal hippocampal commissure and alveus), which were more numerous in CC002 and CC023 than in CC012xCC032, contributed in some way to differentiating the “delayed reflex” profile from “resilience” profile.

We further connected lesion locations and frequencies to genetic background to better understand potential reasons underlying different lesion and phenotypic profiles. Lesion frequency in the hippocampal adjacent regions were significantly correlated with a 200 kilobase region on chromosome 13 which harbors 4 genes. Two of these genes are listed as “predicted” at this time (Gm26043 and Gm30836) and could potentially encode long non-coding RNA genes with roles in regulating the expression and activity of other genes. The other 2 genes, nidogen 1 (*Nid1*) and lysosomal trafficking regulator (*Lyst*), have been confirmed to participate in viral-induced pathology. *Lyst* has an immunoregulatory role on toll-like receptor (TLR) pathways, particularly proinflammatory responses of TLR3 and TLR4 [55]. Mutations in the *Lyst* gene were associated with hemophagocytic lymphohistiocytosis following Epstein-Barr infection of a human patient [56]. It is therefore conceivable *Lyst* may also have a role in TMEV inflammation and lesion development. *Nid1*, on the other hand, is known to be adversely affected by cytomegalovirus infection in humans in such a way as to obliterate the normal functions of the nidogen 1 protein. The viral infection caused down-regulation of the nidogen 1 gene and degradation of the protein it encodes: a basement membrane protein crucial for the integrity of vascular walls [57]. The result was increased transmigration and reduced vascular integrity, allowing the cytomegalovirus to disseminate further. These outcomes, if recapitulated in TMEV-infected hosts, could potentially open the door for lesion development.

Our findings add to the growing body of evidence showing that neurological sequelae are usually the product of more than one lesion, and that lesions can affect individuals differently based on other factors such as genetic background. This supports the concept of network models, in which several regions/contributing factors contribute to overall phenotypic profile. Even in TMEV infection of SJL mice, lesion load is not the sole contributing factor to deficits such as impaired rotarod performance [58]. Additionally, some lesions may be considered “silent” as has been documented extensively for MS [59]. Furthermore, similar lesions may affect mice phenotypically in different ways based on sex, perhaps via compensatory mechanisms and/or hormonal activities. Such effects have been well documented (e.g. in MS; [60]). In the current study we observed no sex differences in lesion number or location; however, we previously identified significant sex differences in phenotypes and disease progression [15]. Therefore, we cannot rule out the possibility of finding more subtle differences in lesions following further analysis with immunohistochemistry or other imaging methods (e.g. electron microscopy).

Future investigations will focus on understanding viral tropism and spread in CC strains across both the acute and chronic phases of infection. Moreover, by characterizing earlier histological responses to infection, we aim to better understand the mechanisms of lesion formation and development. Future extensive analysis of CC strains within different disease subtypes may aid in elucidating how genetic variation influences TMEV susceptibility as well as the morphological changes to the CNS and provide a more thorough understanding of the variation in pathophysiology in response to TMEV infection.

## Supporting information

S1 Fig Difference between sham and infected 90 dpi cumulative clinical phenotype frequency for each CC and inbred mouse strain. Difference between sham and infected 90 dpi cumulative clinical phenotype frequency for each strain. C57BL/6J and SJL/J are presented here as comparators. Data shown here are mean ± SEM of the average clinical phenotype frequency across 90 dpi for each strain. CC002, n = 6 sham, n = 7 infected; CC012xCC032, n = 8 sham, n = 12 infected; CC023, n = 7 sham, n = 9 infected; CC027, n = 6 sham, n = 9 infected; CC057, n = 6 sham, n = 7 infected; CC078, n = 2 sham, n = 4 infected; C57BL/6, n = 5 sham, n = 8 infected; SJL/J, n = 6 sham, n = 7 infected.
(TIFF)

S2 Fig Cumulative clinical phenotype frequency of neurological and sickness phenotypes between sexes for each CC and inbred mouse strain. Differences between female and male 90 dpi cumulative phenotype frequencies for each strain. C57BL/6J and SJL/J are presented here as comparators. Data shown here are mean ± SEM of the average clinical phenotype frequency across 90 dpi for each strain. CC002, n = 4 female, n = 3 male; CC012xCC032, n = 5 female, n = 7 male; CC023, n = 5 female, n = 4 male; CC027, n = 4 female, n = 5 male; CC057, n = 3 female, n = 4 male; CC078, n = 2 female, n = 2 male, C57BL/6, n = 4 female, n = 4 male; SJL/J, n = 3 female, n = 4 male.
(TIFF)

S3 Fig Cumulative clinical phenotype of neurological and sickness phenotypes of traditionally used inbred mouse strains C57BL/6J and SJL/J infected with TMEV. Cumulative frequency of neurological and sickness phenotypes, based on observations over a 90-day period, varied by strain. Data shown here are mean ± SEM of the average clinical phenotype frequency across 90 dpi for each strain. C57BL/6, n = 8 infected; SJL/J, n = 7 infected.
(TIFF)

S4 Fig Clinical progression of neurological and sickness phenotypes varied by CC and inbred mouse strains infected with TMEV. For each phenotype, positive progression scores indicate that severity increased over time. C57BL/6J and SJL/J are presented here as comparators. Data shown here are mean ±SEM of the progression score for each strain. CC002, n = 7 infected; CC012xCC032, n = 12 infected; CC023, n = 9 infected; CC027, n = 9 infected; CC057, n = 7 infected; CC078, n = 4 infected; C57BL/6, n = 8 infected; SJL/J, n = 7 infected.
(TIFF)

S5 Fig Clinical progression of neurological and sickness phenotypes varied by strain and between sham and infected CC and inbred mice. For each phenotype, positive progression scores indicate that severity increased over time. C57BL/6J and SJL/J are presented here as comparators. Data shown here are mean ± SEM of the progression score for each strain. CC002, n = 6 sham, n = 7 infected; CC012xCC032, n = 8 sham, n = 12 infected; CC023, n = 7 sham, n = 9 infected; CC027, n = 6 sham, n = 9 infected; CC057, n = 6 sham, n = 7 infected; CC078, n = 2 sham, n = 4 infected; C57BL/6, n = 5 sham, n = 8 infected; SJL/J, n = 6 sham, n = 7 infected.
(TIFF)

S6 Fig Clinical progression of neurological and sickness phenotypes varied by sex across CC and inbred mouse strain. There were no significant sex differences in clinical progression. For each phenotype, positive progression scores indicate that severity increased over time. C57BL/6J and SJL/J are presented here as comparators. Data shown here are mean ± SEM of the progression score for each strain. CC002, n = 4 female, n = 3 male; CC012xCC032, n = 5 female, n = 7 male; CC023, n = 5 female, n = 4 male; CC027, n = 4 female, n = 5 male; CC057, n = 3 female, n = 4 male; CC078, n = 2 female, n = 2 male, C57BL/6, n = 4 female, n = 4 male; SJL/J, n = 3 female, n = 4 male.
(TIFF)

S7 Fig QTL analysis and relationship to hippocampal adjacent regions. QTL analysis using gQTL identified a region on mouse chromosome 13 which was significantly associated with lesion frequency at the hippocampal-adjacent regions (“Adj”). Panel A shows the significant peak at chromosome 13; note the tall sub-peaks were a product of the small sample size. The associated SNP on chromosome 13 was highly significant. Panel B provides a closer view of the associated region surrounding chromosome 13 SNP UNC22144316, as well as the 4 genes located in the region. The contributions of CC founder strains are also shown.
(TIF)

S8 Fig Cross-sections of the cerebrum at level B of C57BL/6J mice infected with Theiler’s Murine Encephalomyelitis Virus (TMEV) and euthanized ~90 dpi. A. Infected mouse: Complete loss of CA1 neurons with collapse of the stratum oriens, multifocal perivascular mononuclear infiltrate, gliosis, and scattered mineralization. Hematoxylin and eosin stain; bar = 100 μm. B. Sham mouse: Normal hippocampus for comparison. Hematoxylin and eosin stain; bar = 200 μm.
(TIF)

S9 Fig SJL/J mice infected with Theiler’s Murine Encephalomyelitis Virus (TMEV) and euthanized at ~90 dpi. A and B. Cross section of the brain at the level D: Locally extensive demyelination of the white-gray matter interface with marked gliosis and multifocal perivascular lymphocytic infiltrate. Hematoxylin and eosin stain; bar = 100 μm. C and D. Cross section of the thoracic spinal cord: Unilateral demyelination at the interface of the ventral horn and ventral and lateral funiculi with axonal degeneration, gliosis, and lymphocytic meningitis. Hematoxylin and eosin stain; bar = 100 μm.
(TIF)

S10 Fig Cross-sections of the lumbosacral spinal cord of CC mice strains infected with Theiler’s Murine Encephalomyelitis Virus (TMEV) and euthanized at ~90 dpi. A. Strain CC002 and B. Strain CC023: Multifocal to coalescing decreased axon numbers in the ventral nerve roots. Immunohistochemistry for neurofilament; bar = 50 μm. C. Strain CC002 and D. Strain CC023: Marked (C) to moderate (D) myelin loss of the ventral nerve roots with multifocal myelinophages. Luxol fast blue-PASH; bar = 20 μm.
(TIF)

## Abbreviations

CNS: Central Nervous System
CC: Collaborative Cross
TMEV: Theiler’s Murine Encephalomyelitis Virus
dpi: days post-infection
H&E: Hematoxylin and Eosin
CA1/CA2/CA3: Hippocampal Subregions 1/2/3
RIX: Recombinant Inbred Intercross
PFU: Plaque Forming Unit
PBS: Phosphate-Buffered Saline
i.p.: Intraperitoneal
