# Supplementary figures and images for: Host genetic diversity drives variable central nervous system lesion distribution in chronic phase of Theiler’s Murine Encephalomyelitis Virus (TMEV) infection
Source: PLoS One. 2021 Aug 20;16(8):e0256370. doi: 10.1371/journal.pone.0256370 (PMC8378701; doi:10.1371/journal.pone.0256370)

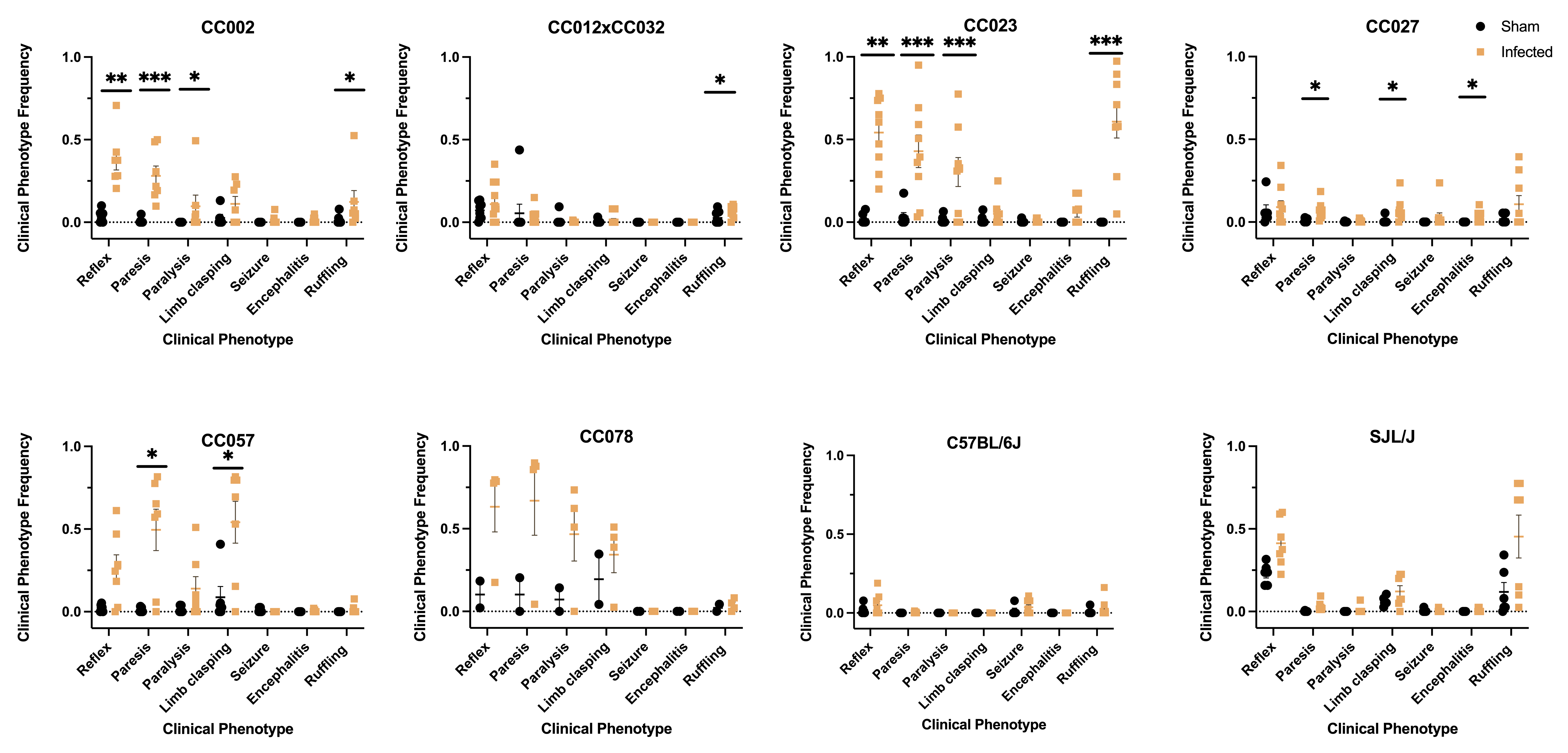

Supplement: S1 Fig — Difference between sham and infected 90 dpi cumulative clinical phenotype frequency for each strain. C57BL/6J and SJL/J are presented here as comparators. Data shown here are mean ± SEM of the average clinical phenotype frequency across 90 dpi for each strain. CC002, n = 6 sham, n = 7 infected; CC012xCC032, n = 8 sham, n = 12 infected; CC023, n = 7 sham, n = 9 infected; CC027, n = 6 sham, n = 9 infected; CC057, n = 6 sham, n = 7 infected; CC078, n = 2 sham, n = 4 infected; C57BL/6, n = 5 sham, n = 8 infected; SJL/J, n = 6 sham, n = 7 infected. (TIFF) [file pone.0256370.s001.tiff]

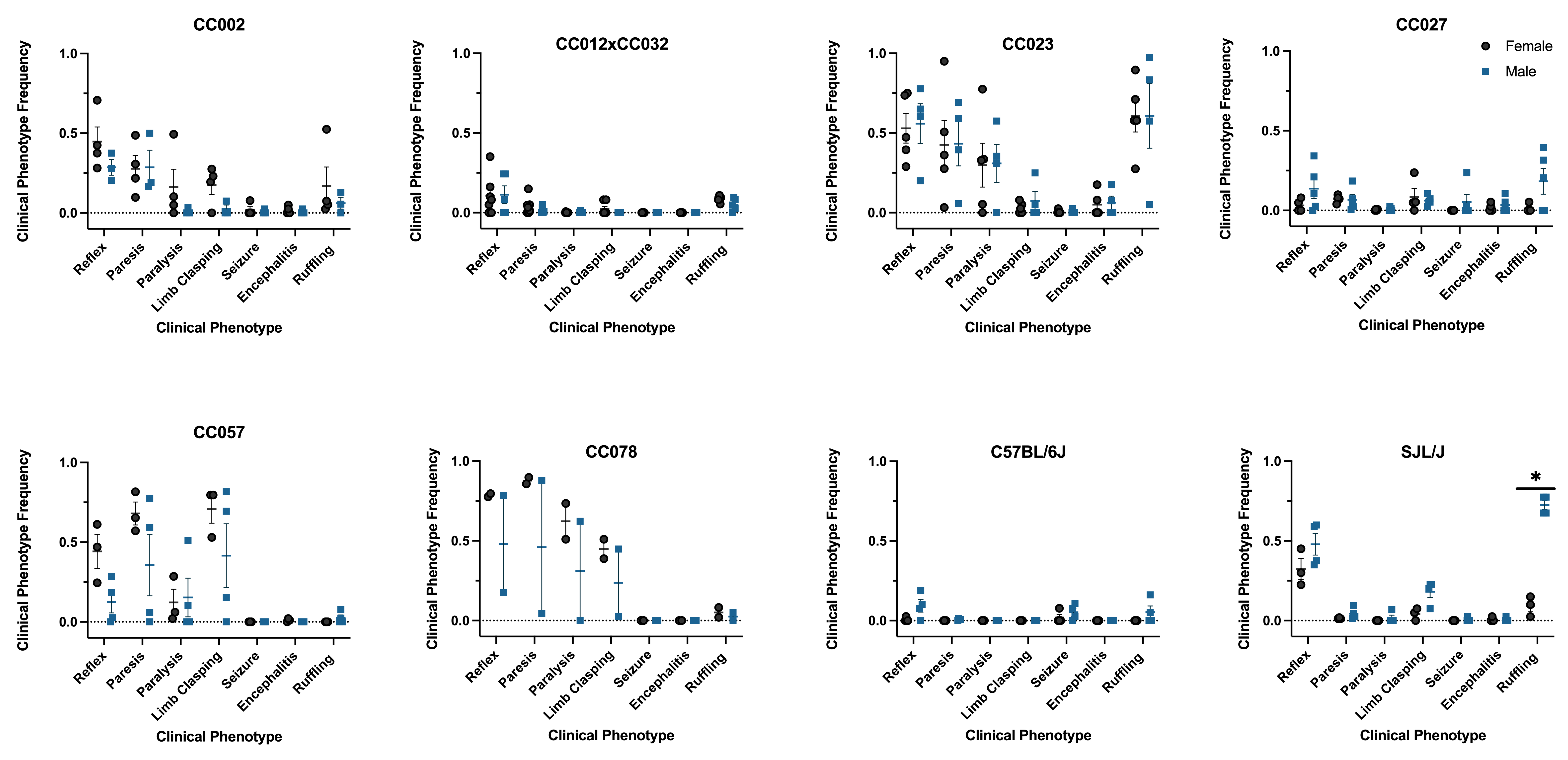

Supplement: S2 Fig — Differences between female and male 90 dpi cumulative phenotype frequencies for each strain. C57BL/6J and SJL/J are presented here as comparators. Data shown here are mean ± SEM of the average clinical phenotype frequency across 90 dpi for each strain. CC002, n = 4 female, n = 3 male; CC012xCC032, n = 5 female, n = 7 male; CC023, n = 5 female, n = 4 male; CC027, n = 4 female, n = 5 male; CC057, n = 3 female, n = 4 male; CC078, n = 2 female, n = 2 male, C57BL/6, n = 4 female, n = 4 male; SJL/J, n = 3 female, n = 4 male. (TIFF) [file pone.0256370.s002.tiff]

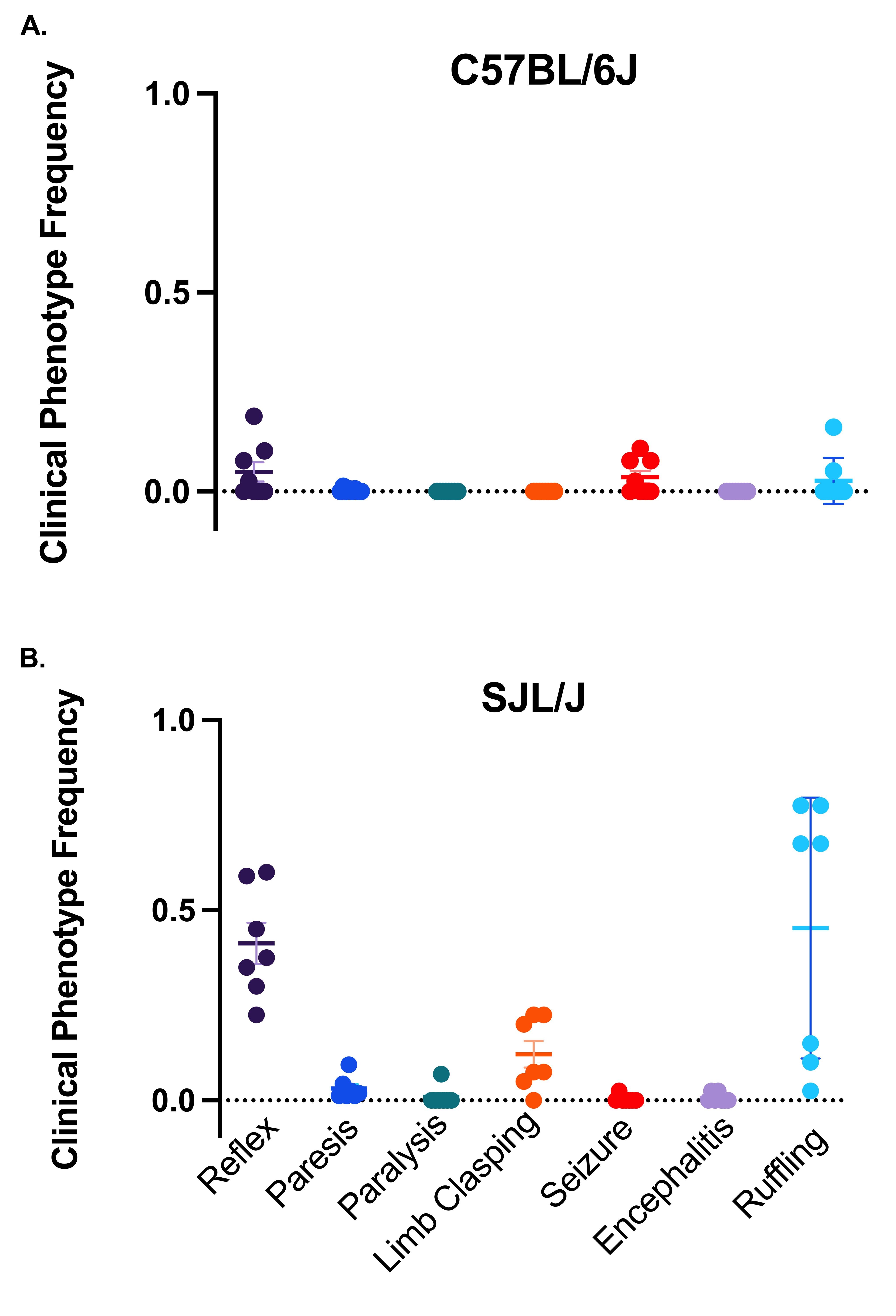

Supplement: S3 Fig — Cumulative frequency of neurological and sickness phenotypes, based on observations over a 90-day period, varied by strain. Data shown here are mean ± SEM of the average clinical phenotype frequency across 90 dpi for each strain. C57BL/6, n = 8 infected; SJL/J, n = 7 infected. (TIFF) [file pone.0256370.s003.tiff]

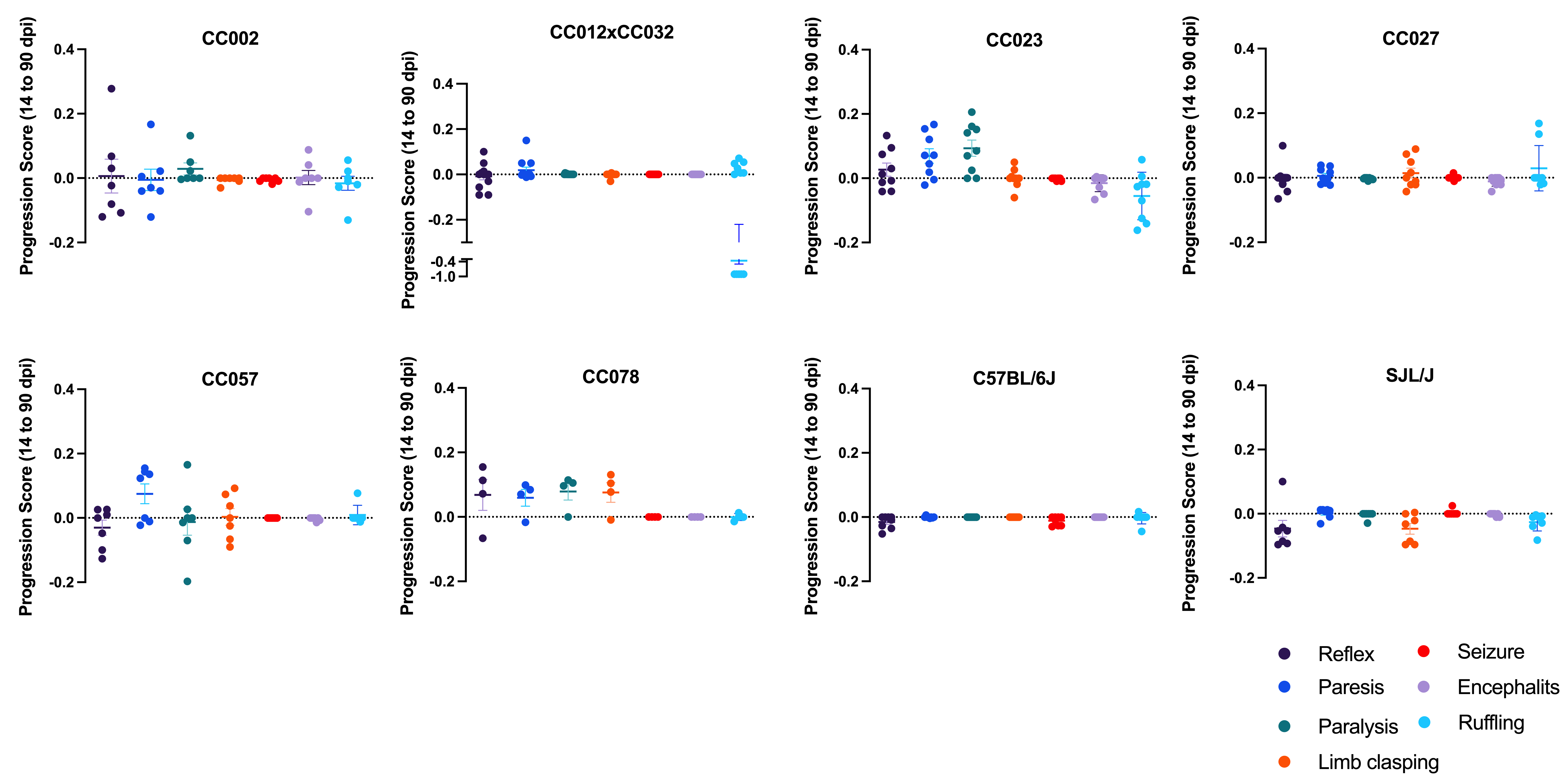

Supplement: S4 Fig — For each phenotype, positive progression scores indicate that severity increased over time. C57BL/6J and SJL/J are presented here as comparators. Data shown here are mean ±SEM of the progression score for each strain. CC002, n = 7 infected; CC012xCC032, n = 12 infected; CC023, n = 9 infected; CC027, n = 9 infected; CC057, n = 7 infected; CC078, n = 4 infected; C57BL/6, n = 8 infected; SJL/J, n = 7 infected. (TIFF) [file pone.0256370.s004.tiff]

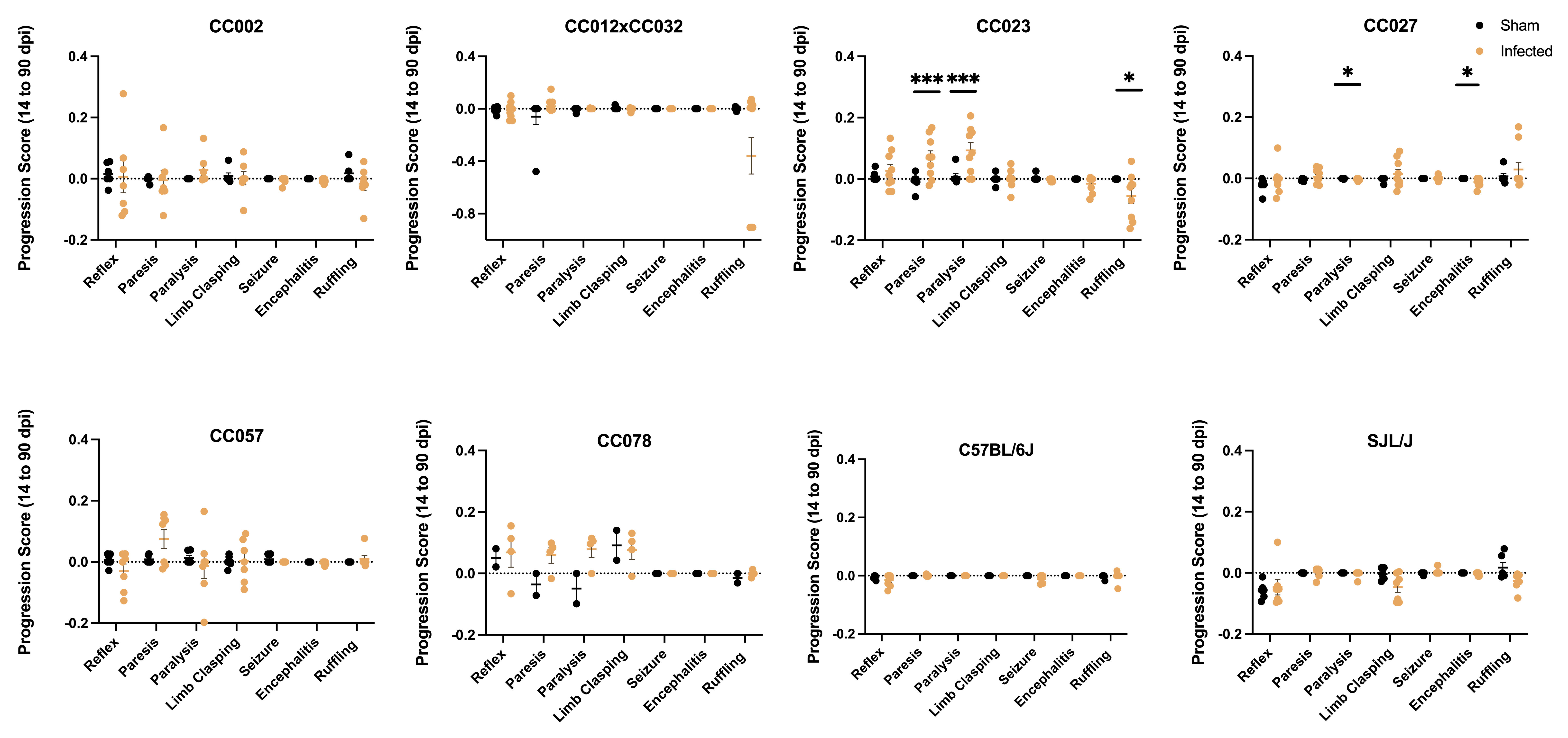

Supplement: S5 Fig — For each phenotype, positive progression scores indicate that severity increased over time. C57BL/6J and SJL/J are presented here as comparators. Data shown here are mean ± SEM of the progression score for each strain. CC002, n = 6 sham, n = 7 infected; CC012xCC032, n = 8 sham, n = 12 infected; CC023, n = 7 sham, n = 9 infected; CC027, n = 6 sham, n = 9 infected; CC057, n = 6 sham, n = 7 infected; CC078, n = 2 sham, n = 4 infected; C57BL/6, n = 5 sham, n = 8 infected; SJL/J, n = 6 sham, n = 7 infected. (TIFF) [file pone.0256370.s005.tiff]

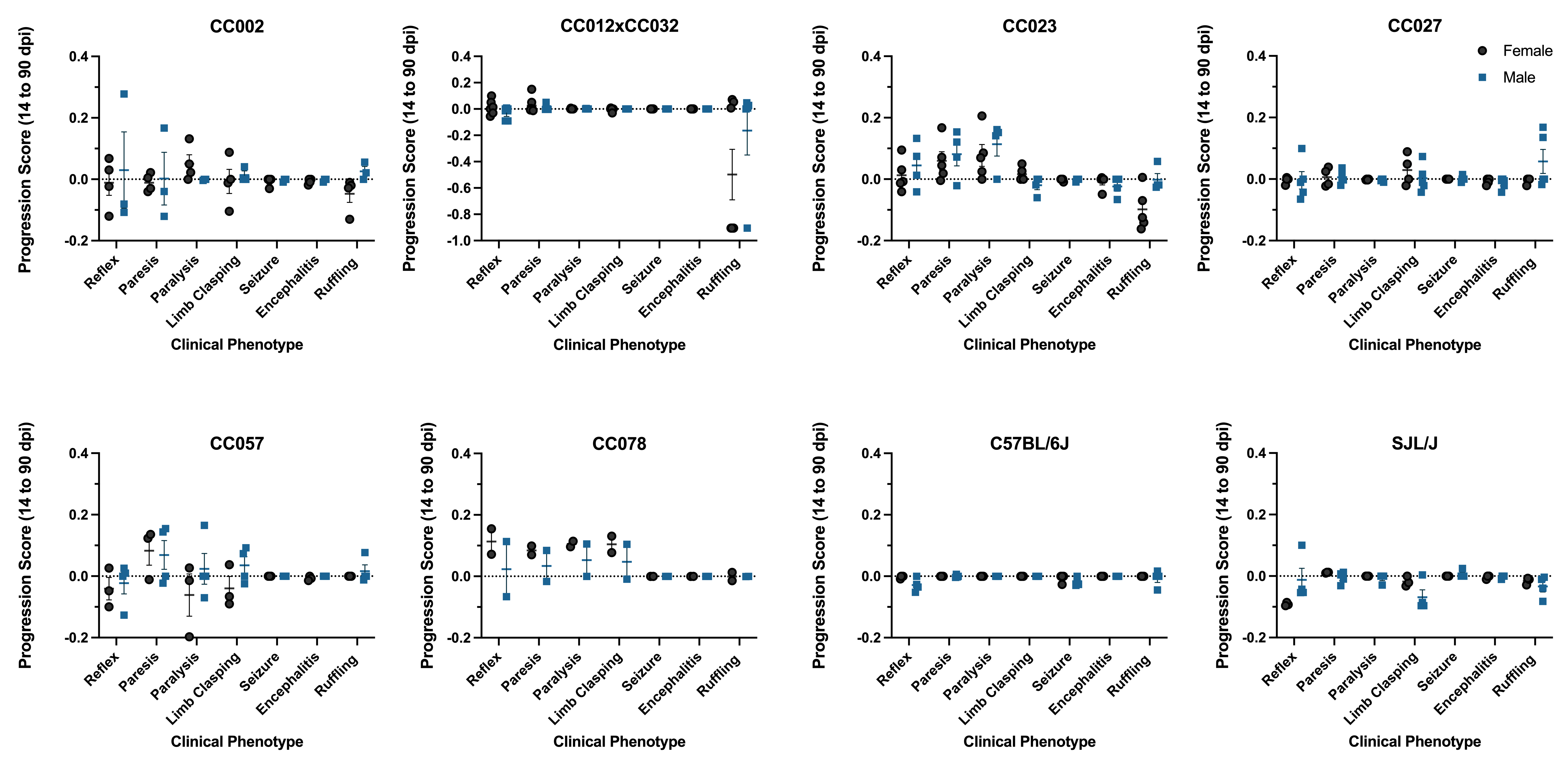

Supplement: S6 Fig — There were no significant sex differences in clinical progression. For each phenotype, positive progression scores indicate that severity increased over time. C57BL/6J and SJL/J are presented here as comparators. Data shown here are mean ± SEM of the progression score for each strain. CC002, n = 4 female, n = 3 male; CC012xCC032, n = 5 female, n = 7 male; CC023, n = 5 female, n = 4 male; CC027, n = 4 female, n = 5 male; CC057, n = 3 female, n = 4 male; CC078, n = 2 female, n = 2 male, C57BL/6, n = 4 female, n = 4 male; SJL/J, n = 3 female, n = 4 male. (TIFF) [file pone.0256370.s006.tiff]

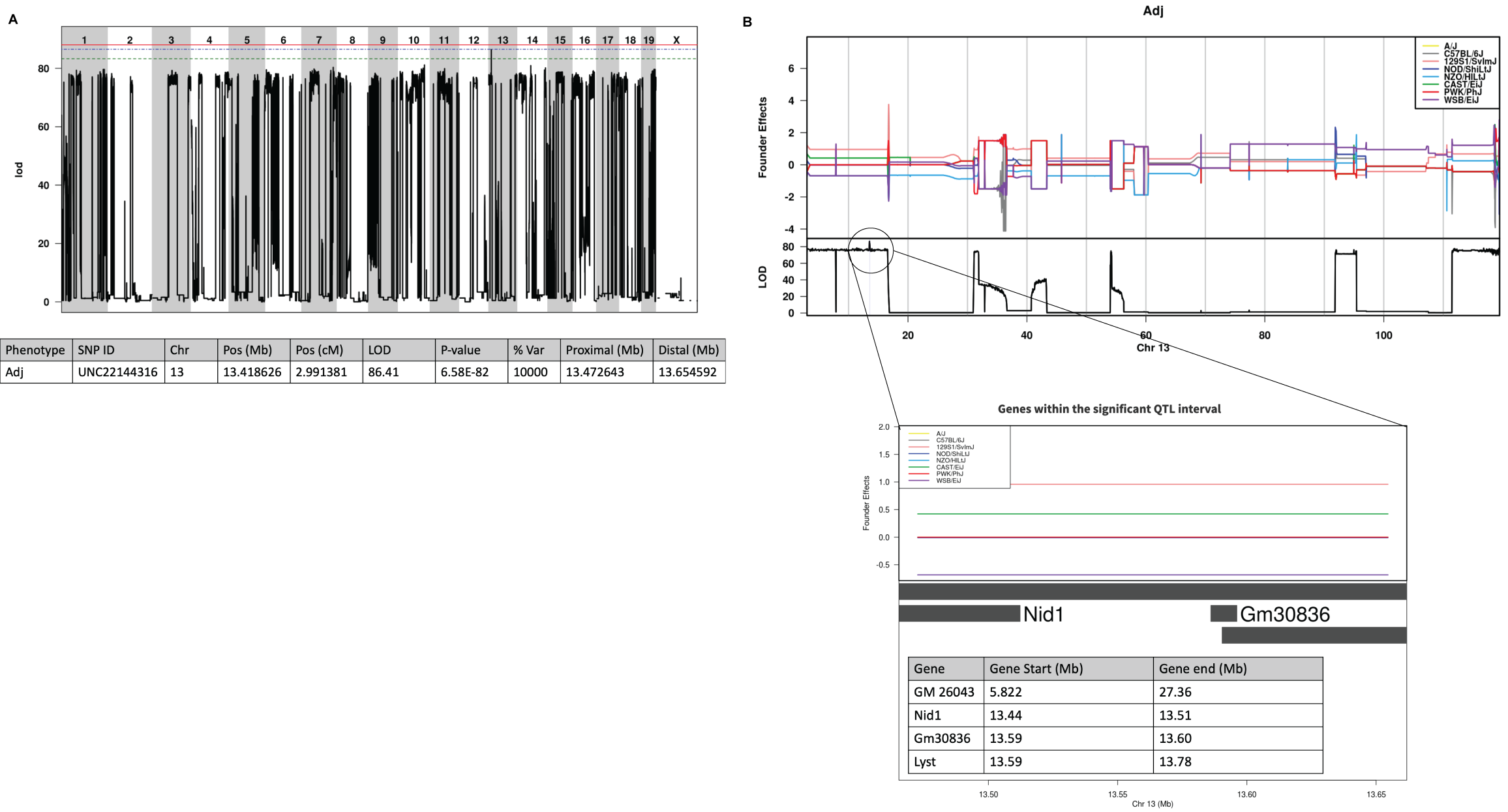

Supplement: S7 Fig — QTL analysis using gQTL identified a region on mouse chromosome 13 which was significantly associated with lesion frequency at the hippocampal-adjacent regions (“Adj”). Panel A shows the significant peak at chromosome 13; note the tall sub-peaks were a product of the small sample size. The associated SNP on chromosome 13 was highly significant. Panel B provides a closer view of the associated region surrounding chromosome 13 SNP UNC22144316, as well as the 4 genes located in the region. The contributions of CC founder strains are also shown. (TIF) [file pone.0256370.s007.tif]

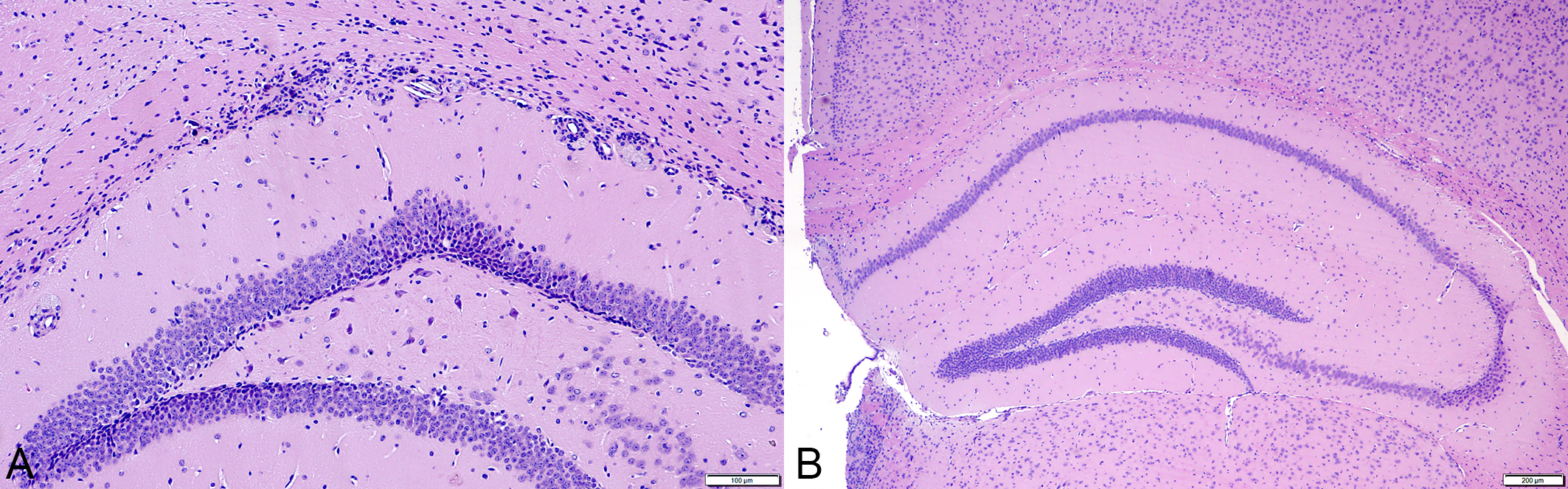

Supplement: S8 Fig — A. Infected mouse: Complete loss of CA1 neurons with collapse of the stratum oriens, multifocal perivascular mononuclear infiltrate, gliosis, and scattered mineralization. Hematoxylin and eosin stain; bar = 100 μm. B. Sham mouse: Normal hippocampus for comparison. Hematoxylin and eosin stain; bar = 200 μm. (TIF) [file pone.0256370.s008.tif]

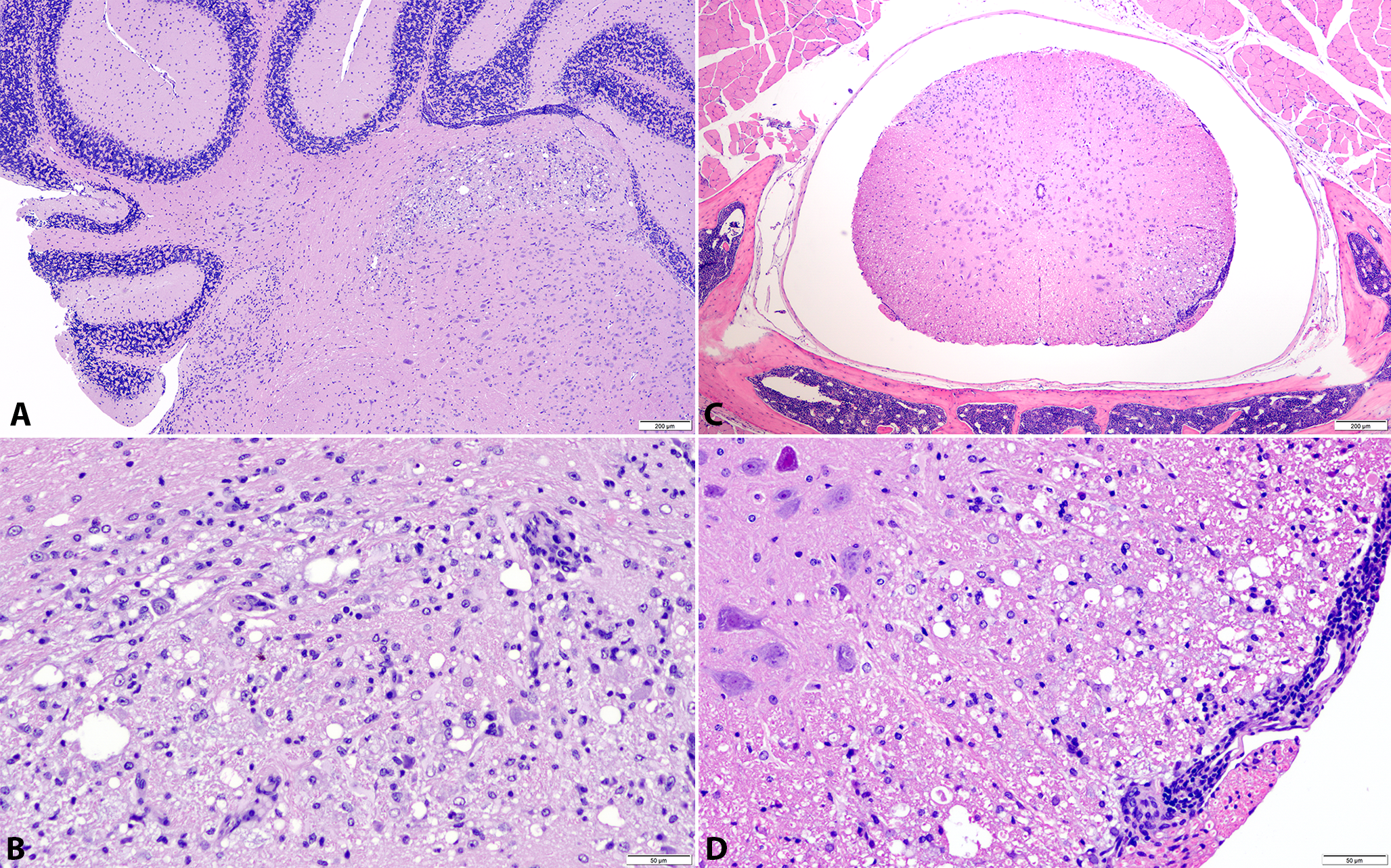

Supplement: S9 Fig — A and B. Cross section of the brain at the level D: Locally extensive demyelination of the white-gray matter interface with marked gliosis and multifocal perivascular lymphocytic infiltrate. Hematoxylin and eosin stain; bar = 100 μm. C and D. Cross section of the thoracic spinal cord: Unilateral demyelination at the interface of the ventral horn and ventral and lateral funiculi with axonal degeneration, gliosis, and lymphocytic meningitis. Hematoxylin and eosin stain; bar = 100 μm. (TIF) [file pone.0256370.s009.tif]

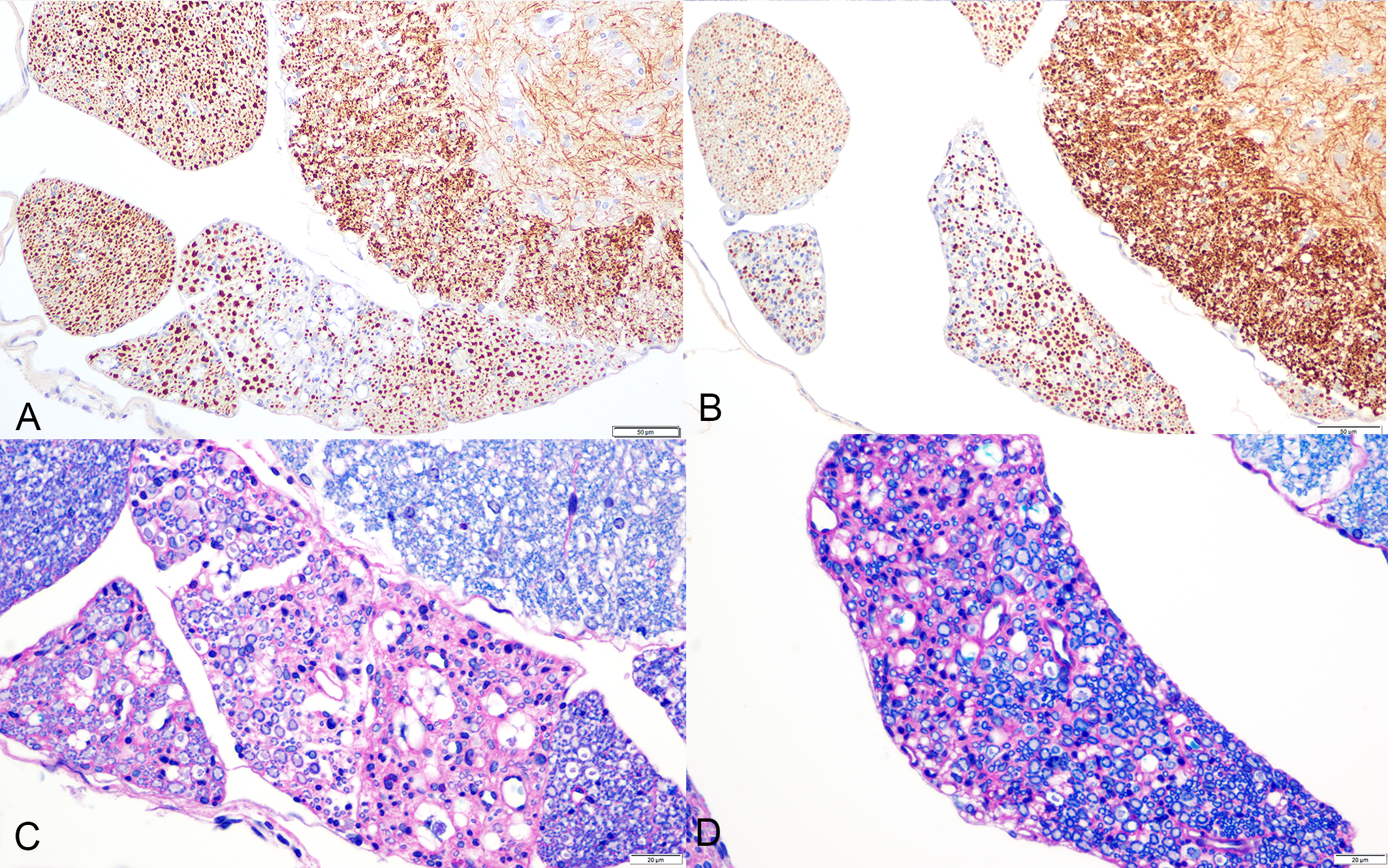

Supplement: S10 Fig — A. Strain CC002 and B. Strain CC023: Multifocal to coalescing decreased axon numbers in the ventral nerve roots. Immunohistochemistry for neurofilament; bar = 50 μm. C. Strain CC002 and D. Strain CC023: Marked (C) to moderate (D) myelin loss of the ventral nerve roots with multifocal myelinophages. Luxol fast blue-PASH; bar = 20 μm. (TIF) [file pone.0256370.s010.tif]
